# A Depth-Adaptive Waveform Decomposition Method for Airborne LiDAR Bathymetry

**DOI:** 10.3390/s19235065

**Published:** 2019-11-20

**Authors:** Shuai Xing, Dandi Wang, Qing Xu, Yuzhun Lin, Pengcheng Li, Lin Jiao, Xinlei Zhang, Chenbo Liu

**Affiliations:** 1 The Institute of Geospatial Information, Strategic Support Force Information Engineering University, 62 Science Road, Zhengzhou 450001, China; wdd_93@163.com (D.W.); 13937169139@139.com (Q.X.); lyz120218@163.com (Y.L.); lpclqq@163.com (P.L.); xd_jiao@foxmail.com (L.J.); zxl9602@163.com (X.Z.); quantum.lcb1994@gmail.com (C.L.); 2 Science and Technology on Near-surface Detection Laboratory, 160 Tonghui Road, Wuxi 214035, China

**Keywords:** airborne LiDAR bathymetry, waveform decomposition, signal detection, waveform classification, deconvolution

## Abstract

Airborne LiDAR bathymetry (ALB) has shown great potential in shallow water and coastal mapping. However, due to the variability of the waveforms, it is hard to detect the signals from the received waveforms with a single algorithm. This study proposed a depth-adaptive waveform decomposition method to fit the waveforms of different depths with different models. In the proposed method, waveforms are divided into two categories based on the water depth, labeled as “shallow water (SW)” and “deep water (DW)”. An empirical waveform model (EW) based on the calibration waveform is constructed for SW waveform decomposition which is more suitable than classical models, and an exponential function with second-order polynomial model (EFSP) is proposed for DW waveform decomposition which performs better than the quadrilateral model. In solving the model’s parameters, a trust region algorithm is introduced to improve the probability of convergence. The proposed method is tested on two field datasets and two simulated datasets to assess the accuracy of the water surface detected in the shallow water and water bottom detected in the deep water. The experimental results show that, compared with the traditional methods, the proposed method performs best, with a high signal detection rate (99.11% in shallow water and 74.64% in deep water), low RMSE (0.09 m for water surface and 0.11 m for water bottom) and wide bathymetric range (0.22 m to 40.49 m).

## 1. Introduction

Airborne LiDAR bathymetry (ALB) is a technique for measuring the depths of moderately clear, near-shore coastal waters and lakes with a high-powered, high-pulsed green laser from a low-altitude aircraft (200–500 m above ground level (AGL)) [1,2,3]. This technique can provide high-density, high-accuracy, and three-dimensional bathymetric data safely and with high efficiency compared with shipborne sonar. The swath width of an ALB system roughly ranges from 0.5–0.75 times AGL, namely 100–400 m, indicating that the area covered within 1 h ranges from about 20–60 km^2^ [4]. Since ALB is cost-effective and time-saving, it has been widely used in coastal mapping, sediment budgets and seabed protection [5,6].

Since the transmission of green laser involves air and water, the waveform processing for the ALB system sometimes could be challenging. Water absorption and scattering influence the extraction of the water surface and bottom returns from the waveform of a green channel, especially for turbid water and shallow water (a depth lower than 0.5 m) [7,8]. Water column scattering, which is much more powerful than atmospheric scattering, leads to a low signal-to-noise ratio (SNR) in the received waveform. Two main problems in the full waveform processing for ALB are given below:

1. Mixed peaks in surface return: The waveforms are the convolution of the emitted pulse and the target cross section, and are digitized by the receiver. The limited full width at half maximum (FWHM) of the emitted pulse and sampling rate in the LiDAR digitizer induce the peak stretching, leading to a mixed peak of surface return and water column scattering. Especially when the water is extremely shallow, the bottom return will also be included in the mixed peak. Taking the mixed peak as the surface return may introduce errors ranging from 10 cm to 25 cm [9].
2. Weak bottom return in deep or turbid water: The pulse energy decreases exponentially with depth in the water column, and the decrease rate is positively associated with water turbidity, resulting in a rather weak bottom return in deep or turbid water [10].

For the first existing problem, some ALB systems use an NIR channel or a Raman channel to measure the surface return. Pe’eri and Philpot [11] studied the relationship between the shape of the Raman waveform and the water depth and used the Raman channel to measure the water depths (shallower than 2 m). However, this algorithm may vary with water clarity. Allouis et al. [12] found that the Raman signal is not reliable for depth estimation, since the Raman signal is sensitive to water characteristics and proposed a depth estimation method using NIR and green fitted waveforms. Another alternative is to establish a water surface model to determine the position deviation between the mixed peak and the water surface, so that the single green laser ALB systems can be used to estimate the water depth. Mandlburger et al. [9] analyzed the near water surface penetration (NWSP) properties of green laser signals. Zhao et al. [13] studied the factors that influence the NWSP of a green laser and proposed an NWSP modeling method. This method needs an auxiliary NIR laser and some SSC sampling stations, which sometimes are difficult to obtain, and so waveform decomposition may be the optimal solution to this problem. For topographic LiDAR, the Gaussian model is sufficient for most applications [14]. However, the Gaussian decomposition method is not suitable for ALB because the water column component in the waveform cannot be easily fitted by Gaussian functions [15,16]. The triangular function [17] and quadrilateral function [18] were both introduced to water column fitting but were only verified by simulated data. Ding et al. [19] proposed an improved quadrilateral model for the water column fitting which shows a better fit to the field data compared with the quadrilateral function. For very shallow water, a surface-volume-bottom (SVB) algorithm was proposed by Schwarz et al. [20] and was applied to measure a riverbed. However, waveforms at different depths vary greatly, and a single model is only suitable for waveforms in a specific depth range.

For the second problem, the key is detecting the bottom signal from low-SNR waveforms. Numerous waveform processing methods have been developed for waveform de-noising or signal enhancement. Saylam et al. [21] used a moving-average filtering algorithm for waveform smoothing. Pan et al. [7] introduced a continuous wavelet transformation (CWT) to project the signal into a continuous time and scale subspace and found that more signals can be detected from the reconstructed waveform while many signals are still undetectable. Wang et al. [15] examined some waveform processing methods and concluded that the Richardson–Lucy deconvolution (RLD) [22] is good at dealing with waveforms with a very shallow depth and a weak bottom response, and the average square difference function (ASDF) [23] can better cope with noise. Richter et al. [24] proposed an attenuation correction procedure to improve the detectability of water bottom signals. Launeau et al. [25] proposed a waveform processing method including smoothing and edge enhancing to increase the detection rate of the bottom signal in moderately turbid water. The de-noising and signal-enhancing methods can improve the detection rate, but the detection accuracy is still limited by the waveform sampling interval, which may also require waveform decomposition. 

Although the previous methods have partially solved the above problems, there is no single best waveform processing strategy across all applications [7,26]. The received waveform influenced by the water depth and the optical attenuation of the water column is varied and complicated. If each component of the waveform is reconstructed, then both the water surface and bottom signals can be precisely detected, and the accurate water depth can be estimated. In this study, we proposed a depth-adaptive waveform decomposition method for green channel waveforms of ALB. Waveforms are first classified by depth based on the shape. In waveform decomposition, two models are developed for waveforms with different depths based on the calibration waveform and an improved quadrilateral model, and a trust region algorithm is introduced to solve the model parameters. Field data from an airborne topo-bathymetric LiDAR system and simulated data are used to assess the accuracy of the detected water surface and bottom positions. The rest of the paper is organized as follows. The field datasets used in this paper are introduced in Section 2, followed by the detailed description of the proposed wave decomposition method. The experimental results are presented and discussed in Section 3. Finally, Section 4 concludes this paper.

## 2. Materials and Methods

### 2.1. The Mapper5000 System and Study Area

The field data were collected by an airborne topo-bathymetric LiDAR (Mapper5000) system in the Qilianyu Islands, Hainan Province, China. This ALB system was developed by the Shanghai Institute of Optics and Fine Mechanics, Chinese Academy of Science. The system performed an elliptical scan at a scan angle of ±15° giving a field of view (FOV) of 30° over a range of 16.8 km × 2.2 km, and the detailed parameters are shown in Table 1. The sensor consists of four channels, including one NIR channel (1064 nm, avalanche photodiode (APD)) and three green channels (532 nm, photomultiplier tube (PMT)). These three green channels are set differently. PMT1 and PMT2 have a shallow field angle, while PMT3 has a wide field angle. The instantaneous field of view (IFOV) of NIR, PMT1 and PMT2 is 6 mrad, and the IFOV of PMT3 is 6‒40 mrad. The receiving directions of PMT1 and PMT2 are perpendicular to each other. Because signals in PMT1 waveforms are generally stable, this study focuses on the processing of PMT1 waveforms, and waveforms in other channels are used as references. Besides this, the calibration waveforms collected in the laboratory are utilized as the approximation of the emitted signal. With the detected bathymetric signals, point clouds can be generated by a data processing software. This data processing software is designed for the Mapper5000 system including geo-calibration and refraction correction for the point clouds. Detailed descriptions of the Mapper5000 system and its point clouds generation software are appended in Appendix A and Appendix B, respectively.

**Figure 1:**
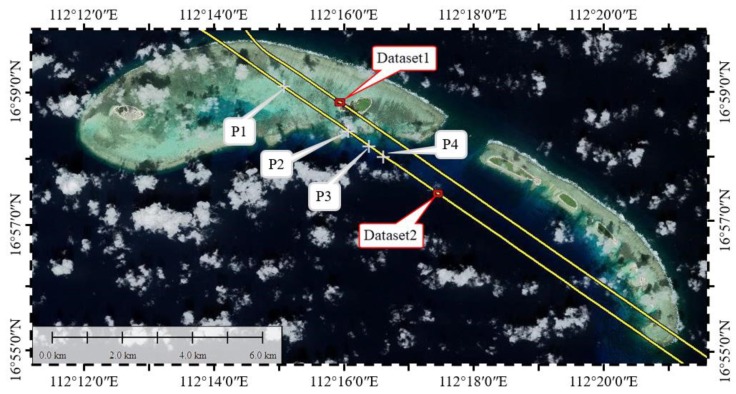
Study area. The yellow solid lines denote the distribution of the strips, the range of the datasets are in the red rectangles, and “+” denote the positions of the bathymetric points (P1–P4).

For the statistical analysis of waveforms, we choose four bathymetric points (P1–P4) with different depths. In order to access the performance of the proposed waveform decomposition method, especially in areas where the water depth is deep or shallow, two representative datasets, Dataset 1 and Dataset 2, were selected from two different strips. Figure 1 shows the locations of the strips, the datasets and bathymetric points in the study area.

### 2.2. Workflow

**Figure 2:**
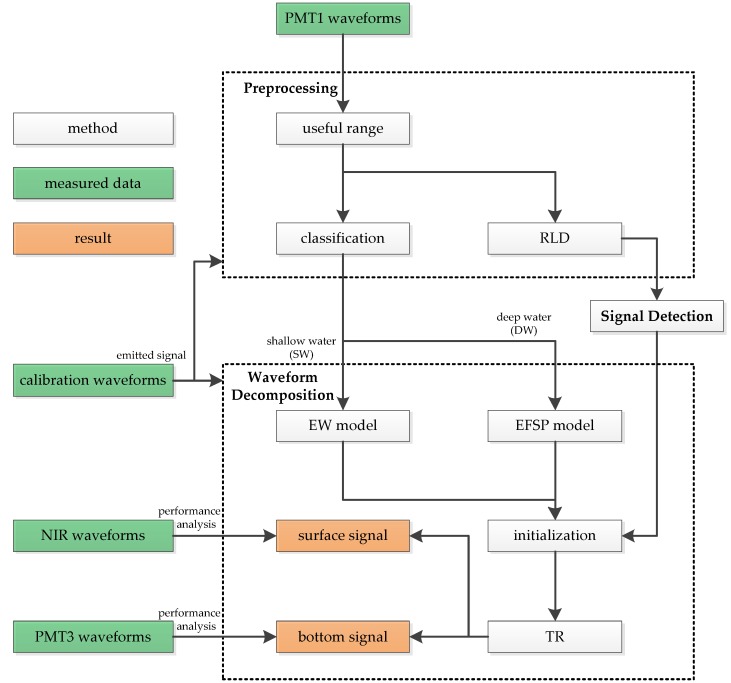
Workflow of the depth-adaptive waveform decomposition method. PMT: photomultiplier tube; RLD: Richardson–Lucy deconvolution; EFSP: exponential function with second-order polynomial model; EW: empirical waveform model; NIR: near-infrared; TR: trust region algorithm.

The original PMT1 waveforms are processed with the depth-adaptive waveform decomposition method to extract the accurate positions of the bathymetric signals. As shown in Figure 2, this is a multistep process including preprocessing, signal detection and waveform decomposition. The first step was to determine the useful range of the waveforms, classify the waveforms by depth and improve the signal resolution by RLD. After deconvolution, the second step was to detect the signals with an adaptive threshold, and the results were used as the initial values of the following waveform decomposition. PMT1 waveforms were classified into two categories, “shallow water (SW)” and “deep water (DW)”. In the last step, the fitting model was selected based on the waveform category. With the initial values provided by the second step, the model parameters were solved by the TR algorithm. After waveform decomposition, the surface and bottom signals were extracted from the waveforms. To evaluate the accuracy of the results, signals detected from NIR waveforms and PMT3 waveforms were used as reference data.

### 2.3. Preprocessing

#### 2.3.1. Useful Range

To ensure the ALB system can measure both land and water, the system normally records thousands of samples for the received waveform, while the signals only exist in 0.8–5% of it. Thus, if the range of the signals, which is known as the “useful range”, can be determined, the waveform processing will be more efficient. Jutzi and Stilla [27] proposed a criterion that if the waveform is three times higher than the noise power for at least 5 ns, a signal will be assumed to have been found. In this paper, we choose the last 10% of the waveform to estimate the noise. According to the maximum measurable depth and flight height of the system, signals cannot exist in this range. The truncation noise *N_T_* and the noise power *N_P_* can be estimated using the minimum amplitude and the standard deviation of the waveform in this range, respectively. The noise level *N_L_* can be expressed as follows:

*N_L_* = *N_T_* + 3*N_P_*. (1)

**Figure 3:**
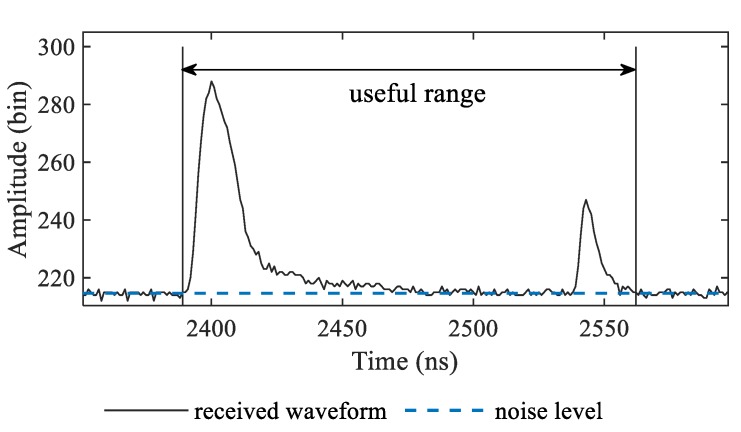
The useful range of the received waveform. The received waveform recorded by the ALB system ranges from 0–3200 ns, while the useful range is 2389–2562 ns.

By searching the waveform using a criterion that a waveform higher than *N_L_* and lasting for no less than 5 ns may indicate a signal, the useful range can be determined as shown in Figure 3.

#### 2.3.2. Waveform Classification

**Figure 4:**
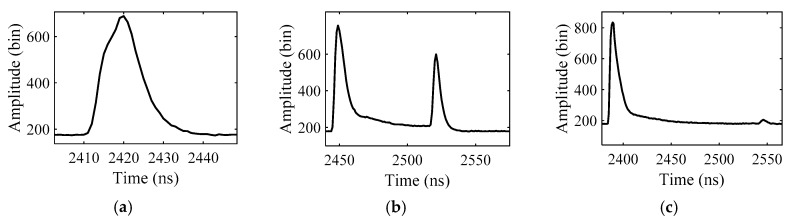
The received waveforms with depths of (**a**) 0.6 m, (**b**) 10.8 m and (**c**) 23.9 m.

Received waveforms of the green LiDAR are varied with depths as shown in Figure 4. To fit the waveforms with different depths, waveforms are grouped into two categories, “shallow water” and “deep water”, according to a defined parameter *S*. Because the depths of the water that results in the surface and bottom signal overlap vary with different ALB systems or measured area, it is difficult to classify waveforms with an absolute water depth threshold. Based on the waveform analysis, we found that the shape of the water column scattering in the received waveform is almost fixed in a small measured area (See Section 3.1.1). If the water column scattering can be captured in the waveform, it also indicates that there is no overlap between the water surface and the bottom signal. Thus, the water column scattering can be used as a sign to classify the waveforms. In this study, shallow water and deep water are not distinguished by absolute water depth but by waveform shape. If the surface and bottom signal overlap in the waveform, it is defined as “SW”; otherwise, the waveform is labeled as “DW”.

**Figure 5:**
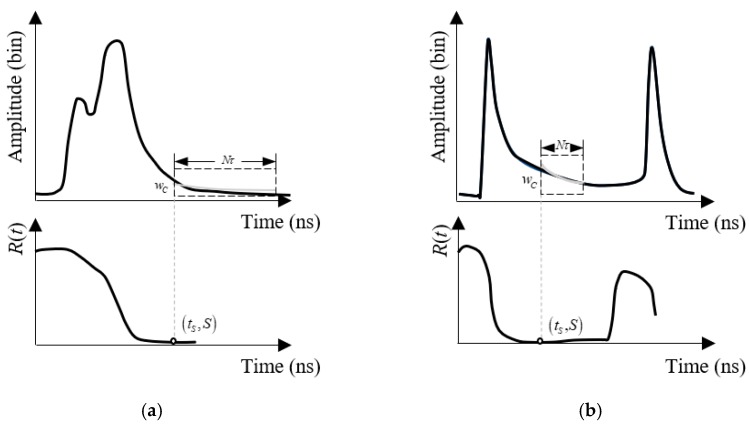
*R*(*t*) of (**a**) a shallow water (SW) waveform with a large *S* of 4220.5 and (**b**) a deep water (DW) waveform with a small *S* of 133.4.

The parameter *S* is determined by the following equations:

(2) S=min{R(t)},

(3) $$R\left(t\right)=\frac{1}{N}\sum_{m=1}^{N}{\left[w_{C}\left(m\tau \right)-w_{R}\left(m\tau +t\right)\right]}^{2},$$

where *w_R_* is the received waveform, *w_C_* is a section of the water column scattering truncated from the received waveforms, *N* is the sampling number of *w_C_*, and *τ* is the sampling interval of the system. Because the received waveforms in extremely deep water have complete water column scattering (see Figure 4c), *w_C_* can be easily obtained from them. Specific details are given in Section 3.1.1. For the “SW” waveform, the water column scattering is embedded by surface and bottom signals, and so the received waveform is less similar to *w_C_*, as shown in Figure 5a. Because water column reflectance varies little in a small measured area, the “DW” waveform will have a small *S*, as shown in Figure 5b. Thus, received waveforms in which *S* is less than a threshold (*T_S_*) are classified as “DW”, while the remaining waveforms are “SW”.

#### 2.3.3. Richardson–Lucy Deconvolution

Waveforms are processed by RLD to increase the signal resolution. Considering that most of the waveforms are likely to have mixed peaks, especially for the first peak which may contains surface and column return, this overlap mostly results from a convolution of the emitted waveform and the target cross section. Thus, a deconvolution process is needed before detecting the signals.

RLD is an iterative deconvolution method which was developed by Richardson [28] and Lucy [29] to recover a blurred image with a known point spread function (PSF). Many studies [22,30,31] have applied this to LiDAR waveform processing in the time domain to estimate the target cross section, where the emitted waveform is regarded as a PSF. The *i*th iteration can be calculated as

(4) $${\bar{p}}^{i+1}\left(t\right)={\bar{p}}^{i}\left(t\right)\cdot \left(w_{T}\left(t\right)*\frac{w_{R}\left(t\right)}{\left(w_{T}\left(t\right)*{\bar{p}}^{i}\left(t\right)\right)}\right),$$

where “∗” is the convolution product, *w_T_*(*t*) is the emitted waveform which is approximated by the calibration waveform in this paper, *w_R_*(*t*) is the received waveform, and $\bar{p}{\left(t\right)}^{i+1}$ is the *i*th iteration solution of the target cross section. The initial value of *p*(*t*) for the iteration is simply set as *w_R_*(*t*), which has negligible effect on the result. Determining the end point of the iteration is the key of RLD because the noise amplification is due to overfitting. The stopping criterion of RLD used here is detailed discussed in [32].

**Figure 6:**
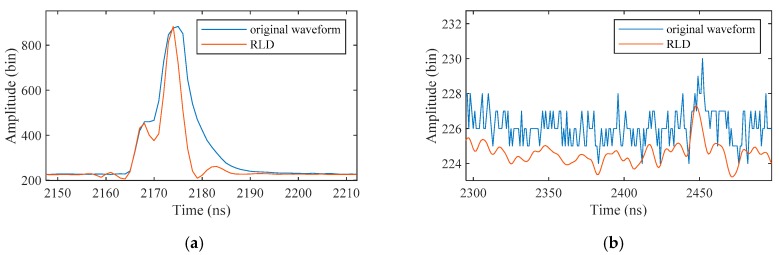
Examples of the deconvoluted waveforms by RLD in (**a**) shallow water and (**b**) deep water. RLD: Richardson–Lucy Deconvolution. The result of the deep water waveform is only displayed in the portion near the bottom signal.

Figure 6 shows that RLD can improve the signal resolution whether in shallow water or deep water waveforms. In shallow water waveforms, RLD can shorten the FWHM of the surface and bottom signals (see Figure 6a). In deep water waveforms, RLD can partially remove the background noise while keeping the weak bottom signal (see Figure 6b). 

### 2.4. Signal Detection

**Figure 7:**
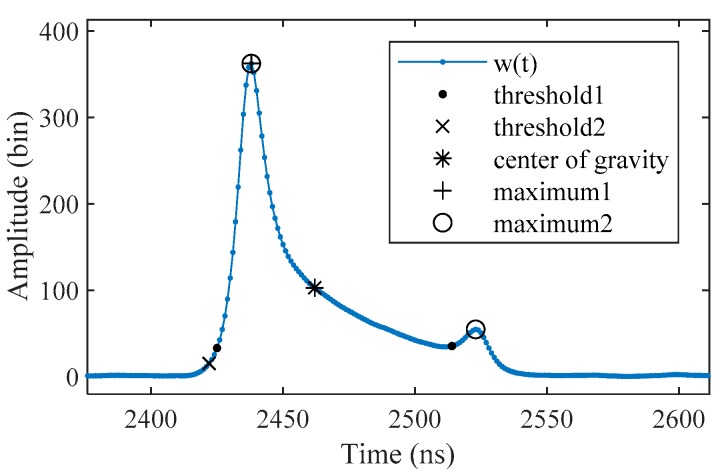
Comparison of the conventional signal detection methods, where “*threshold1*” used a threshold that was equal to one-tenth of the maximum amplitude of *w_R_*(*t*) (*M_A_*), “*threshold2*” used a threshold that was equal to 0.05 *M_A_*, “*maximum1*” used a threshold that was equal to 0.2 *M_A_*, and “*maximum2*” used a threshold that was equal to 0.1 *M_A_*.

A number of signal detection methods such as *threshold*, *center of gravity* and *maximum* are often used in conventional LiDAR waveform processing [33]. However, most of them may not work for *w_R_*(*t*) in ALB. We tested some of the conventional signal detection methods, as shown in Figure 7. It could be determined that the *center of gravity* method may not be suitable for ALB signal detection, due to the component of water column scattering in the received waveform. The *threshold* and *maximum* methods can give more reliable results with an appropriate threshold. However, the appropriate threshold may vary in different waveforms.

**Figure 8:**
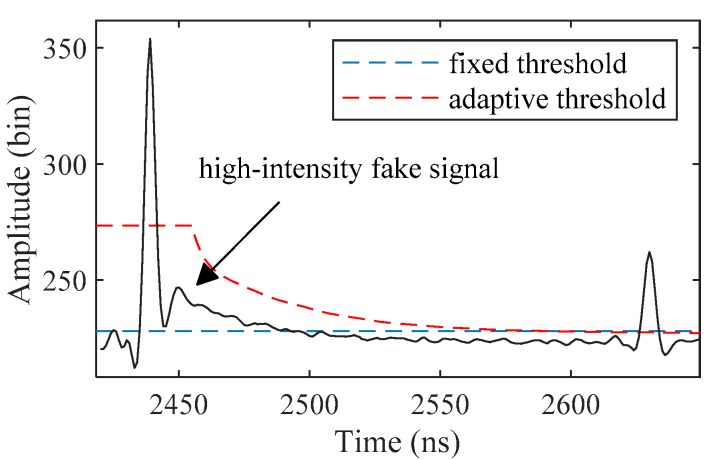
Thresholds used in signal detection. The high-intensity fake signals are located in water column scattering, which may be detected by the *maximum* method with a fixed threshold.

In this section, an adaptive threshold was used for ALB signal detection instead of a fixed one, as shown in Figure 8. The adaptive threshold *T*(*t*) is defined as

(5) $$T\left(t\right)=\left\{ \begin{array}{l} \max\left\{w_{C}\right\}+3\times N_{P},\;t<t_{S}\\ w_{C}\left(t-t_{S}\right)+3\times N_{P},\;t\geq t_{S} \end{array}\right.,$$

where *t_S_* is the minimum point corresponding to the minimum value *S* of *R*(*t*). As the water depth changes, *T*(*t*) will be adjusted reasonably, effectively avoiding the influence of high-intensity fake signals on signal detection and improving the reliability of the results.

Candidate signals in deconvoluted waveforms are detected with the *maximum* method and filtered by *T*(*t*). If there are more than two remaining signals, the two signals with the largest amplitude are retained.

### 2.5. Waveform Decomposition

There are three basic steps in waveform decomposition, including modeling, initialization and fitting. The waveform processed here is denoted by *w*(*t*), which is the original received waveform truncated at noise level *N_L_*. 

#### 2.5.1. Modeling

The key to waveform decomposition is to build a reasonable model. Some models for the ALB waveform have been introduced, such as two Gaussian functions [12], a combination of a Gaussian function, a triangle function and a Weibull function [17], a combination of two Gaussian functions and a quadrilateral function [18], and a chain of exponential segments [34]. Considering that water depth is an important factor influencing the shape of waveform (as shown in Figure 4), two models are proposed for “SW” and “DW” waveforms, respectively.

The fitting model *f_W_*(*t*) can be expressed as

(6) $$f_{W}\left(t\right)=f_{S}\left(t\right)+f_{B}\left(t\right)+f_{C}\left(t\right).$$

**Figure 9:**
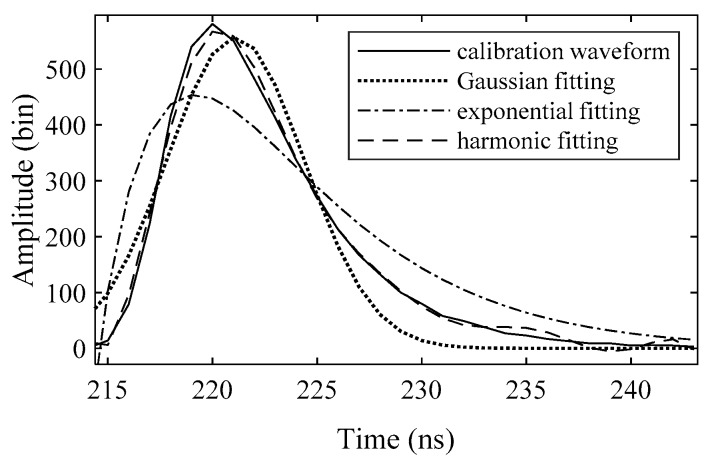
Calibration waveform (solid) and fitting curves (dashed) with Gaussian, exponential and harmonic models. The Gaussian model includes only one Gaussian function. The exponential model is a sum of two exponential functions, and the harmonic model is a combination of four harmonic functions. All fitting parameters were solved by a Levenberg–Marquardt algorithm, and the adjusted R-Square (R^2^) coefficients of Gaussian, exponential and harmonic models are 0.9332, 0.8015 and 0.9956 respectively.

For the surface return model *f_S_*(*t*) and bottom return model *f_B_*(*t*), the existing models use a specific function, such as Gaussian, exponential and harmonic function, to model the surface and bottom return, but are not exact in practice because of the distortion caused by the sensor response, as shown in Figure 9. Thus, we do not use a model with some defined functions, but directly use a transformation *C* of the calibration waveform to better fit the surface and bottom return:

(7) $$C\left(A,\mu ,\sigma ,\varphi \right)=A\varphi \left(\frac{t-\mu }{\sigma }\right),$$

where *A* is the amplitude scaling factor, *μ* is the time shift factor, *σ* is the time scaling factor, and ***φ*** is the normalized calibration waveform. The calibration waveform used here can be collected in the laboratory or substituted by a waveform received from bare ground, and ***φ*** is estimated using the smoothing spline method, normalized in amplitude, and shifted in time to locate the peak at the zero point.

The surface return model *f_S_*(*t*) is given by

(8) $$f_{S}\left(t\right)=C\left(A_{S},\mu_{S},\sigma_{S},\varphi \right),$$

where *A_S_*, *μ_S_* and *σ_S_* are the amplitude scaling factor, time shift factor and time scaling factor in function *C*, respectively.

The bottom return model *f_B_*(*t*) is given by

(9) $$f_{B}(t)=C\left(A_{B},\mu_{B},\sigma_{B},\varphi \right),$$

where *A_B_*, *μ_B_* and *σ_B_* are the amplitude scaling factor, time shift factor and time scaling factor in function *C*, respectively.

For “SW” waveforms, the water column scattering model *f_C_*_1_(*t*) is equal to

(10) $$f_{C1}(t)=C\left(A_{C},\mu_{C},\sigma_{C},\varphi \right).$$

where *A_C_*, *μ_C_* and *σ_C_* are the amplitude scaling factor, time shift factor and time scaling factor in function *C*, respectively. For “SW” waveforms, the proposed model *f_W_*(*t*) is based on the calibration waveform and is therefore named the empirical waveform model (EW).

**Figure 10:**
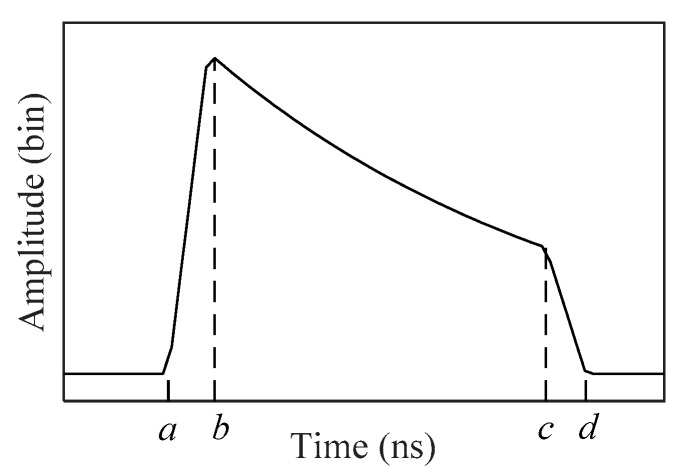
Water column scattering model.

For “DW” waveforms, *f_C_*(*t*) is defined as

(11) $$f_{C2}(t)=\left\{ \begin{array}{ll} \exp\left(fb^{2}+gb+h\right)\left(\frac{t-a}{b-a}\right) & a<t\leq b\\ \exp\left(ft^{2}+gt+h\right) & b<t\leq c\\ \exp\left(fc^{2}+gc+h\right)\left(\frac{d-t}{d-c}\right) & c<t\leq d\\ 0 & else \end{array}\right.,$$

where *a*, *b*, *c* and *d* are the horizontal coordinates of four boundary points in *f_C1_*(*t*), as shown in Figure 10, and *f*, *g* and *h* are coefficients related to water column scattering. Here, an exponential function with a second-order polynomial is proposed to improve the quadrilateral model presented in [19]. Hence, this model is named the exponential function with second-order polynomial model (EFSP).

Thus, the proposed model *f_W_*(*t*) can be denoted by *f_W_*(*t*, ***γ***) with the unknown parameter vector

(12) $$\gamma =\left(A_{S},A_{B},A_{C},\mu_{S},\mu_{B},\mu_{C},\sigma_{S},\sigma_{B},\sigma_{C}\right),$$

or

(13) $$\gamma =\left(A_{S},A_{B},\mu_{S},\mu_{B},\sigma_{S},\sigma_{B},a,b,c,d,f,g,h\right).$$

#### 2.5.2. Initialization

Initialization is a significant process in waveform decomposition. Waveform decomposition is a nonlinear nonnegative least-squares problem, which can be solved by many algorithms, but global convergence cannot be promised, especially when there are a large number of parameters to solve. 

The first step of initialization is to calculate the initial value ***γ***_0_. The results of signal detection in Section 2.4, namely the rough positions of the surface and bottom signals denoted by *t_S_*_0_ and *t_B_*_0_, are used for calculating the initial values of the parameters in ***γ***:

(14) $$\begin{array}{l} \left(A_{S0},A_{B0},A_{C0},\mu_{S0},\mu_{B0},\mu_{C0},\sigma_{S0},\sigma_{B0},\sigma_{C0}\right)=\left(w\left(t_{S0}\right),w\left(t_{B0}\right),0.5\times w\left(t_{B0}\right),t_{S0},t_{B0},0.5\times \left(t_{S0}+t_{B0}\right),1,1,1\right)\\ \left(a_{0},b_{0},c_{0},d_{0}\right)=\left(t_{S0}-0.5\times t_{L},t_{S0}+0.5\times t_{R},t_{B0}-0.5\times t_{L},t_{B0}+0.5\times t_{R}\right), \end{array}$$

where *t_L_* is the length of ***φ*** to the left of the peak, and *t_R_* is the length of ***φ*** to the right of the peak. If only surface signal is detected, the value of *t_B_*_0_ will be set as *t_S_*_0_ + 1/2 × *t_L_*.

In the EFSP model, the last three parameters in ***γ*** are estimated by a simple linear fitting:

(15) $$\begin{array}{c} A_{\mathrm{fit}}=\left[\begin{array}{ccc} \sum t^{4} & \sum t^{3} & \sum t^{2}\\ \sum t^{3} & \sum t^{2} & \sum t\\ \sum t^{2} & \sum t & \sum 1 \end{array}\right]b_{\mathrm{fit}}=\left[\begin{array}{c} \sum \ln\left(w(t)\right)\cdot t^{2}\\ \sum \ln\left(w(t)\right)\cdot t\\ \sum \ln\left(w(t)\right) \end{array}\right]\\ {\left[\begin{array}{ccc} f_{0} & g_{0} & h_{0} \end{array}\right]}^{T}=A_{\mathrm{fit}}{}^{-1}b_{\mathrm{fit}}, \end{array}$$

where the fitting range is set at [*t*_*S*0_ + *t_R_*, *t*_*B*0_ − *t_L_*], because the surface and bottom returns have a negligible effect on this part of the waveform.

#### 2.5.3. Fitting

Regarded as a non-linear least-squares problem, the step of fitting is dealt with by many mature theories and algorithms, such as the Gauss–Newton algorithm (GN), Expectation-Maximization algorithm (EM) [35], Levenberg–Marquardt algorithm (LM) [36,37] and Reversible Jump Monte Carlo Markov Chain (RJMCMC) [38]. However, GN, the most traditional method, easily converges at a local optimal solution, and its modification, LM, although it has a global convergence to some degree, may be influenced by the initial value in practice. Thus, the TR algorithm is introduced in this study. Unlike the other algorithms mentioned above, TR can solve a problem with constraints, indicating that the parameters will be calculated in a reasonable range. Furthermore, TR is not a line search algorithm which is carried out along a search direction in each iteration; instead, TR searches the next iterated point in a trust region, which is a neighborhood of the current iterate point. The principle is described below [39].

The cost function *Q*(***γ***) is expressed as:

(16) $$\min\;Q\left(\gamma \right)=\sum_{i=1}^{n}{\left[w\left(t_{i}\right)-f_{W}\left(t_{i},\gamma \right)\right]}^{2},\;\gamma \in R^{m},$$

where *n* is the number of sampling points in the useful range, and *m* is the number of parameters in ***γ***. At the *k*th iteration, *Q*(***γ***) is expanded in Tailor at the current iteration point ***γ*** ^(*k*)^ with the second-order terms preserved

(17) $$Q\left(\gamma \right)\approx Q\left(\gamma^{\left(k\right)}\right)+\nabla Q{\left(\gamma^{\left(k\right)}\right)}^{T}\left(\gamma -\gamma^{\left(k\right)}\right)+\frac{1}{2}{\left(\gamma -\gamma^{\left(k\right)}\right)}^{T}\nabla^{2}Q\left(\gamma^{\left(k\right)}\right)\left(\gamma -\gamma^{\left(k\right)}\right).$$

We plug ***d*** = ***γ*** − ***γ*** ^(*k*)^ into Equation (17) and get the quadratic form:

(18) $$\varphi_{k}\left(d\right)=Q\left(\gamma^{\left(k\right)}\right)+\nabla Q{\left(\gamma^{\left(k\right)}\right)}^{T}d+\frac{1}{2}d^{T}\nabla^{2}Q\left(\gamma^{\left(k\right)}\right)d.$$

The trust region in the current iteration can be expressed as ‖***d***‖ ≤ *r_k_*, where *r_k_* is the trust region radius. As the range of ***γ*** is given, we can limit the solution in a reasonable range by setting *r_k_*. Thus, the cost function in Equation (16) will be translated into solving the following trust region subproblem:

(19) $$\begin{array}{l} \min\;\varphi_{k}\left(d\right)\\ s.t.\;\|d\|\leq r_{k}. \end{array}$$

By solving Equation (19) using a line searching algorithm, the optimal solution ***d***^(*k*)^ is obtained. The key part of a TR algorithm is how to judge ***d***^(*k*)^; that is, whether to accept the current improvement ***d***^(*k*)^ and how to change the trust domain radius *r_k_*. According to the chosen strategy, the TR method has many forms [40]. This paper only uses one of them; that is, the correctness of ***d***^(*k*)^ is judged according to the ratio of the actual decrease of the function value to the predicted decrease [41]:

(20) $$\rho_{k}=\frac{Q\left(\gamma^{\left(k\right)}\right)-Q\left(\gamma^{\left(k\right)}+d^{\left(k\right)}\right)}{Q\left(\gamma^{\left(k\right)}\right)-\varphi \left(d^{\left(k\right)}\right)}.$$

If *ρ_k_* ≤ 0, then ***d***^(*k*)^ approximation fails, and we let ***γ*** ^(*k*+1)^ = ***γ*** ^(*k*)^. Conversely, if ***d***^(*k*)^ approximation succeeds, we let ***γ*** ^(*k*+1)^ = ***γ*** ^(*k*)^ + ***d***^(*k*)^ and calculate the new trust domain radius *r_k_*_+1_ according to *ρ_k_*. The new starting point ***γ*** ^(*k*+1)^ and the trust region radius *r_k_*_+1_ are re-substituted into Equations (18) and (19). We repeat these steps until the result converges.

## 3. Results and Discussion

### 3.1. Experiment Ⅰ: Waveform Classification

#### 3.1.1. Statistical Analysis of Waveforms with Different Depths

**Figure 11:**
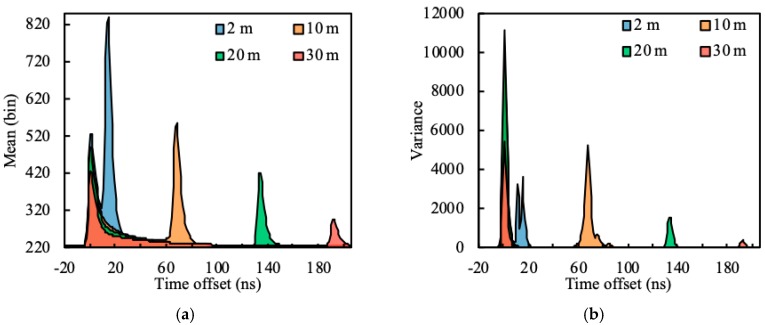
(**a**) Mean curves and (**b**) variance curves of the four bathymetric points. The time offset indicates the offset of the waveform from the peak of the surface signal.

Water depth is one of the most important influence factors of the waveform. To classify the waveform by depth reasonably, waveforms of four bathymetric points (P1–P4) with depths around 2 m, 10 m, 20 m and 30 m are randomly selected for analysis (100 waveforms for each bathymetric point). For waveform registration, each waveform was shifted in time to locate the peak of the surface signal at the zero point. The mean and variance curves of the four bathymetric points were calculated as shown in Figure 11.

As depicted in Figure 11a, the bottom signal is most affected by the water depth, and its amplitude shows an obvious decrease with the increase in water depth. Some differences can also be found in the surface signals due to the change of the measurement time and position. However, the amplitudes of water column scattering are almost constant at different depths. The variance curves in Figure 11b show the difference in waveforms at the same water depth. It can be found that the surface and bottom signals are still changing even at a constant water depth. In contrast, the change in water column scattering is negligible. 

When the water depth is 30 m and the time offset is 10 ns, the variance quickly drops to 60, indicating that the waveform after this point is slightly affected by the surface signal. When the time offset is equal to 30 ns, the amplitude of the mean curves is below 5% of the intensity of the surface signal; this suggests that the shape of water column scattering in the received waveform is almost fixed in a small measured area, which can be used as a sign to classify the waveforms. Therefore, the mean curve with a time offset between 10 ns and 30 ns can be truncated as *w_C_* (see Section 2.3.2).

#### 3.1.2. Distribution of S.

According to the width of the emitted signal, the received waveforms can be divided into three cases:

- Case 1: Water column scattering is completely covered by the surface and bottom signals.
- Case 2: The length of water column scattering that is not covered is less than the length of *w_C_*.
- Case 3: The length of water column scattering that is not covered is greater than the length of *w_C_*.

**Figure 12:**
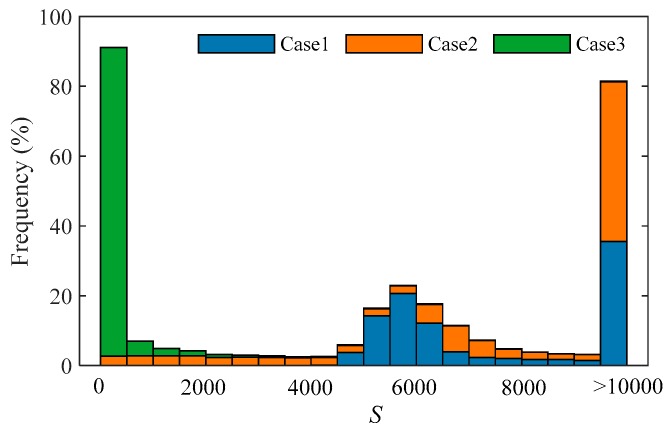
The distribution of *S* in different cases.

In this experiment, waveforms in different cases were selected and *S* is calculated according to Equations (2) and (3). As shown in Figure 12, the *S* values of the waveforms belonging to Case 1 are above 4500, while the *S* values in Case 3 are below 3500. Thus, the threshold *T_S_* can be set between 3500 and 4500. In this paper, *T_S_* is set to 4000.

### 3.2. Experiment Ⅱ: Performance Analysis for the Processing Algorithms in Shallow Water

#### 3.2.1. Reference Data

**Figure 13:**
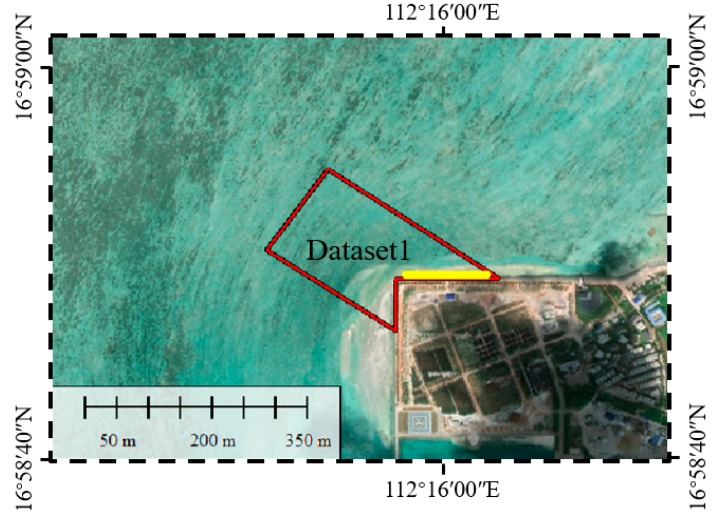
The scope of Dataset 1 (red line) and the selected profile (yellow line).

The bathymetric environment is complex and changeable. Figure 13 shows the scope of Dataset 1. It can be seen that the edge of Dataset 1 is adjacent to the island’s coastline, which is the most affected area by tides. Therefore, in accuracy analysis, the reference data must be acquired simultaneously with the field data. However, the bathymetric sonar is inefficient in shallow waters compared to ALB; it is difficult to ensure these two bathymetric methods are performed simultaneously.

To assess the signal detection accuracy, signals detected from NIR waveforms were taken as references. Because the NIR signal does not penetrate into the water column, it can provide an accurate position of the water surface [9]. For shallow water, its waveform in the PMT1 channel has a strong bottom return. Errors occur only when a mixed peak is detected. Thus, the accuracy of surface signal detection can reflect the accuracy of the water depth.

**Figure 14:**
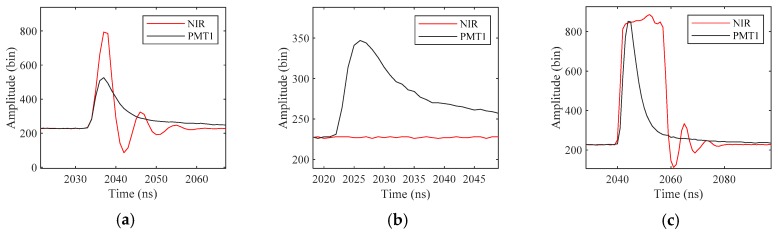
The (**a**) normal, (**b**) buried and (**c**) saturated NIR signals. The NIR signals are not always reliable. Sometimes the surface signal is buried below the noise level which cannot be detected, because its strength is not within the dynamic range of the receiver. Sometimes the surface signal is saturated, because the receiving direction of the sensor is approximately in the reflection direction of sunlight.

However, NIR signals are not always reliable (sometimes buried below the noise level due to strong absorption and sometimes saturated, see Figure 14). In this experiment, NIR signals are filtered according to the signal strength before being used as references.

#### 3.2.2. Surface Signal Detection

To access the performance of the proposed algorithms in shallow water, the traditional *maximum* method was applied to analyze the effect of the adaptive threshold (see Section 2.4). Signals in waveforms processed by RLD are both detected by the *maximum* method with a fixed threshold and adaptive threshold. The corresponding point clouds are referred to as RLD_M and RLD_A. The fixed threshold is set to *N_T_* + 3*N_P_*. The Gaussian model proposed in [12] is used to process waveforms in very shallow water. To compare the applicability of the model, waveforms are both fitted with the Gaussian and EW model. Their initial values are calculated according to RLD_A, and the generated point clouds are referred to as GD (Gaussian decomposition) and EW. The point cloud only processed by the tradition *maximum* method is also presented for comparison (denoted by Max).

**Figure 15:**
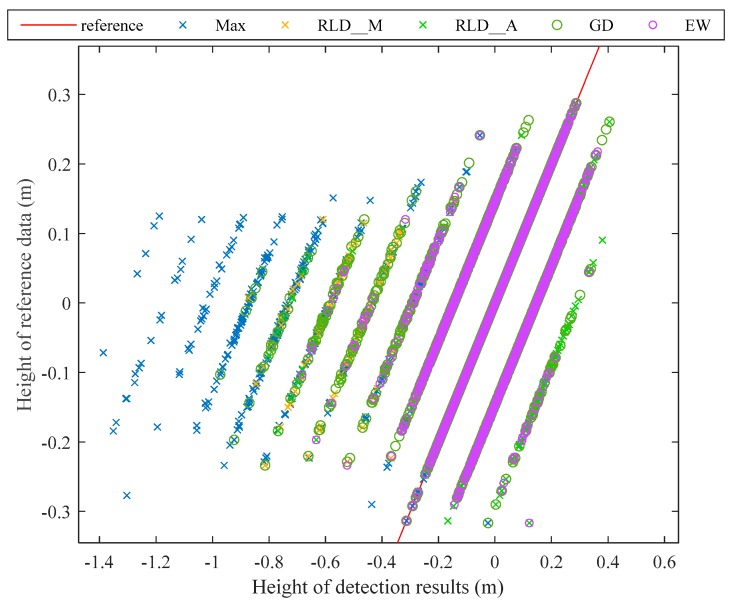
Comparison between the height of the water surface in reference data and detection results.

The water surface points from all the point clouds are compared in height with the reference data (See Figure 15). Most of the inaccurate surface points are lower than the reference data, because the bottom signals are detected as surface signals by mistake or the peak of the surface signal is shifted backward due to the overlap with the bottom signal. The Max is most affected by this problem. Since RLD can improve the signal resolution, this problem has been significantly improved in RLD_M and RLD_A. EW shows a good consistency with the reference data, and most of the errors are within 0.15 m, indicating that the errors of the detected signal position are within one sampling interval of the waveform.

**Figure 16:**
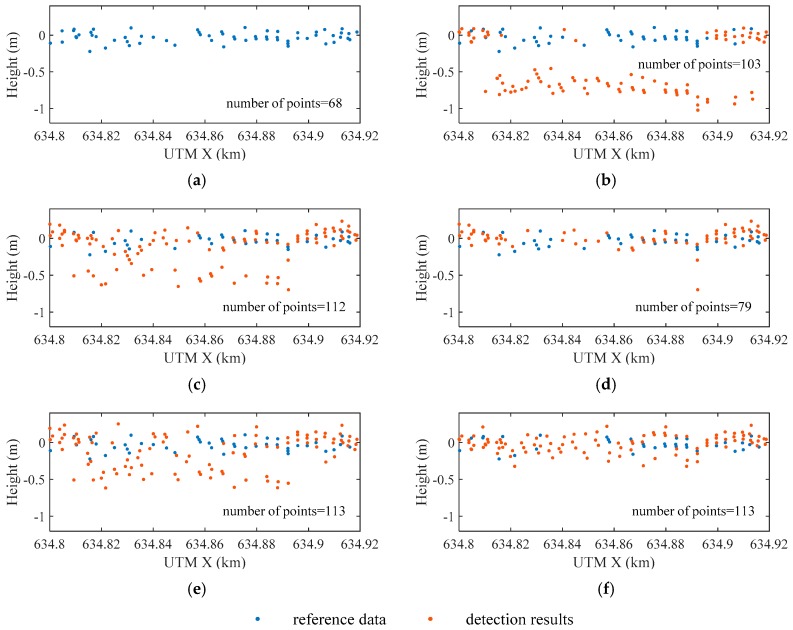
Locations of the water surface on the selected profile from: (**a**) reference data; (**b**) Max; (**c**) RLD_M; (**d**) RLD_A; (**e**) Gaussian decomposition (GD); and (**f**) EW.

Figure 16 shows the water surface on the selected profile (see Figure 13) detected by the above algorithms (only when both surface and bottom signals are detected will the surface point be displayed in this figure). The water depths on this profile are shallower than 1 m. The height of the water surface should float at 0 m, just as with the reference data. The heights of most points in Max are between −1 m and −0.5, indicating that mixed peaks were detected. In RLD_M, more points have been detected, and the number of error points is significantly reduced, proving that RLD can effectively improve the signal resolution. Although the number of points in RLD_A is less than in RLD_M, most of the missing points are outliers. There are still many outliers in GD. Compared with the other results, the distribution of the points in EW is most similar to the reference data, and the difference is no more than 0.5 m.

Table 2 provides the statistical parameters of the above results, where Dr represents the detection rate, which is defined as the percentage of surface points with an error less than 0.3 m; RMSE denotes the root mean square error of the surface points; min(*d*) corresponds to the detected minimum depth; and Std. means the standard deviation of the heights of the surface points.

The detection rate of EW is 99.11%, which is the highest. The detection rate of RLD_M, RLD_A and GD is close, but the result of RLD_A is more reliable, which can be found by comparing RMSE. GD and EW have the minimum min(*d*), followed by RLD_M and RLD_A. Apart from the RMSE, Std. can also reflect the accuracy of signal detection. Since sea level changes very little in a small area, the smaller the Std. is, the more accurate the surface points are. In general, EW performs best, with the highest detection rate, minimum error and maximum bathymetric range. The reference data has a low detection rate (58.54%) and a small Std (0.0839), which indicates that NIR signals are undetectable sometimes but accurate.

#### 3.2.3. Adaptability Analysis of the EW Model

To further study the effects of different models on waveform decomposition, three representative waveforms are selected from Dataset 1. The corresponding depth of the first waveform is more than 1 m, and the initial values provided by RLD_A are exact. The depths of the second and third waveforms are less than 1 m. For the second waveform, the initial values are rough, while for the third waveform, RLD_A only provides the mixed peak position. 

**Figure 17:**
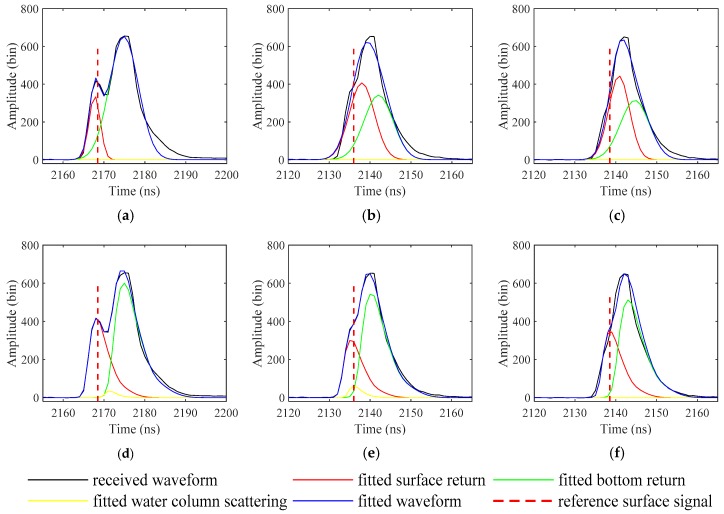
Waveforms fitted with (**a**–**c**) the Gaussian model and (**d**–**f**) the EW model.

Figure 17 shows the fitting results. When the water depth is more than 1 m, the surface returns fitted by the two models are both correct, but the peak position of the surface return fitted by the EW model is closer to the reference data (see Figure 17a,d). When the water depth is less than 1 m, although the whole waveform is well fitted with the Gaussian model, the position of the surface return is not accurate (see Figure 17b,c). In contrast, the EW model is still applicable even in the absence of accurate initial values (see Figure 17e,f). In addition, it can be found that the fitting of water column scattering plays a very small role here, which also follows the practical situation, but it still influences the positions of the fitted surface return and bottom return.

### 3.3. Experiment Ⅲ: Performance Analysis for the Processing Algorithms in Deep Water

#### 3.3.1. Reference Data

**Figure 18:**
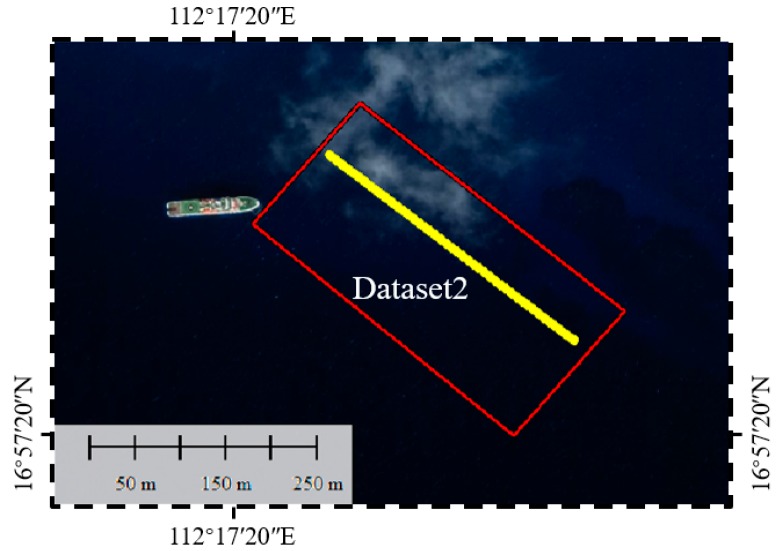
The scope of Dataset 2 (red line) and the selected profile (yellow line).

Dataset 2 is located on a slope with depths ranging from 20 m to 40 m, as shown in Figure 18. This dataset is selected to test the ability of the signal detection algorithms in deep water. The detection of surface signals has been analyzed in shallow water, and for the waveforms in deep water, the difficulty of signal detection mainly lies in the detection of the weak bottom signal. Thus, this experiment focuses on the detection of bottom signals. 

**Figure 19:**
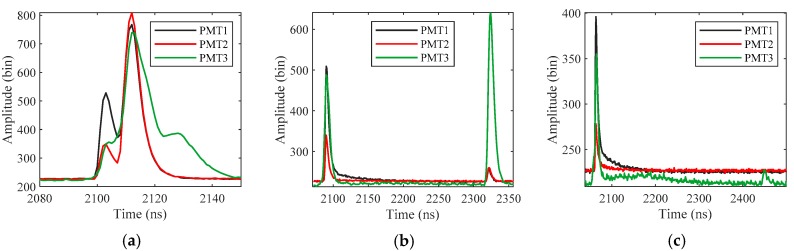
The received waveforms in PMT1, PMT2 and PMT3 with depths of (**a**) 1.4 m, (**b**) 35.1 m and (**c**) 57.8 m.

Here, signals detected from the PMT3 channel are used as reference data. Because PMT3 has a larger field of view than PMT1, the intensity of the bottom return in PMT1 is weaker than that in PMT3 (see Figure 19b). In Dataset 2, the bottom signals are weak in PMT1, but still strong in PMT3. Therefore, we apply the proposed method to PMT1, and the bottom signals detected from PMT3 with the maximum detection method are used as reference data. However, waveforms in PMT3 are not applicable to all cases. When the water is shallow, PMT3 is affected by multiple reflection (see Figure 19a), and when the water depth reaches a certain value, the bottom return in PMT3 is also weak (see Figure 19c). Since the bottom reflection can be approximated as a non-directional diffuse scattering, there is no difference between the bottom return in PMT1 and PMT2. Therefore, the optimal strategy is to process the waveforms in PMT1 when the water is shallow and process the waveforms in PMT3 when the detection of the bottom signal in PMT1 has failed. However, it is still necessary to detect the weak bottom signal in PMT3 effectively. In Dataset 2, the bottom signals in PMT1 are weak, which can simulate the detection of the weak bottom signal in PMT3.

#### 3.3.2. Bottom Signal Detection

In deep water, waveforms may have a weak bottom signal with an amplitude approximately equal to the noise level, which is possibly caused by a deep depth, a turbid water or a dark bottom. Therefore, besides the RLD, which is used to improve the signal resolution, a filtering algorithm which can de-noise the waveform but keep the bottom signal in the meantime is also effective. ASDF is used as a filtering algorithm in [23], which is a substitute for the direct cross-correlation function to estimate the time delay in two discrete time series and is more computationally convenient. ASDF_M and ASDF_A denote the point clouds generated from the signals detected by the *maximum* method with a fixed threshold and the adaptive threshold from the waveforms that are processed by ASDF. We also applied the method proposed in [25] which used a first derivative of a wide Gaussian filter to reduce the processing range of the waveform, and processed the waveforms in this range with normalization, multiple smoothing and three times derivation to increase the detection rate of the bottom signal. The point clouds generated from this method are referred to as dddNCFWF. For the waveform decomposition, since the Gaussian model is no longer applicable, the quadrilateral model (QUAD) introduced in [18] is applied here together with the EFSP model proposed in this paper. These models are initialized with RLD_A, and the corresponding point clouds are referred to as QUAD and EFSP. Before the performance analysis, all the point clouds are filtered according to neighboring points to eliminate outliers.

**Figure 20:**
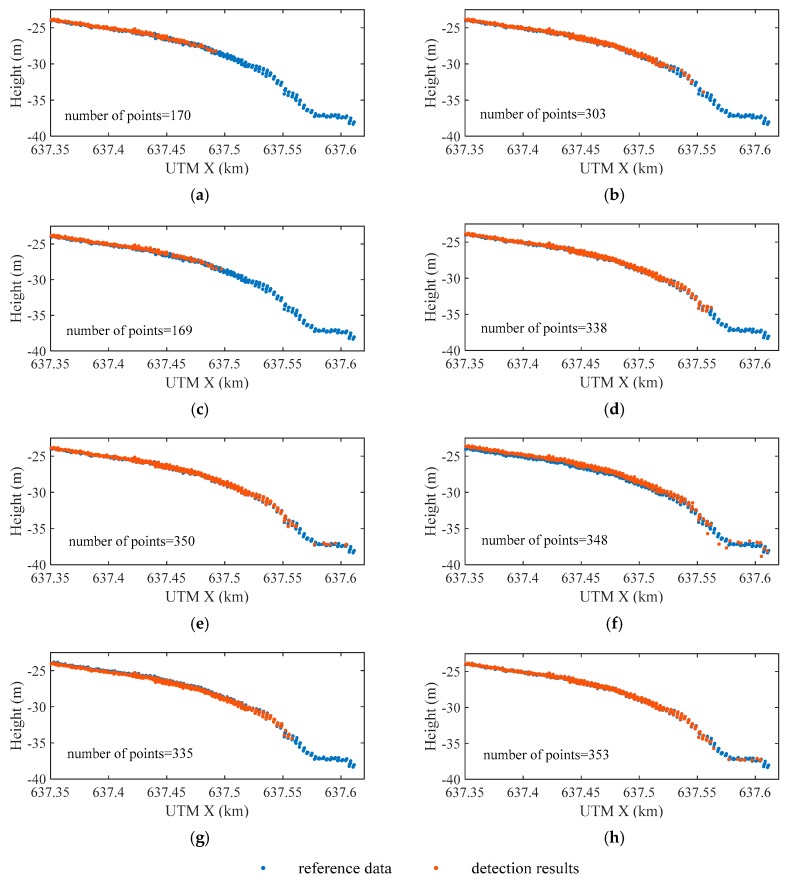
Locations of the water bottom on the selected profile from: (**a**) Max; (**b**) ASDF_M; (**c**) RLD_M; (**d**) ASDF_A (**e**) RLD_A; (**f**) dddNCFWF; (**g**) quadrilateral model (QUAD) and (**h**) EFSP.

Figure 20 shows the detected water bottom on the selected profile (see Figure 18). The number of the detected points in ASDF_M is nearly twice that in Max or RLD_M. However, when the thresholds in the *maximum* method are adaptive, more water bottom points can be detected. RLD_A performs better than ASDF_A, which is exactly the opposite of RLD_M and ASDF_M. The intensity of the bottom signal varies greatly with water depth, but the fixed threshold cannot take all kinds of situations into account. The detection rate of ASDF_M is higher than Max and RLD_M, while the detection rate of ASDF_A is almost equal to that of RLD_A. Because noise can be filtered by ASDF, the results indicate that the fixed threshold is more sensitive to the noise than the adaptive threshold. dddNCFWF provides a high detection rate just as RLD_A. However, there is an offset between it and the reference data which may be induced by the dissimilarity between the emitted waveform and the symmetrical filters used in the method. Even with RLD_A as the initial value, QUAD still cannot accurately fit the waveforms with the depths over 35 m. In contrast, the EFSP model is more flexible, but it seems that there is little difference between RLD_A and EFSP.

In further comparing these algorithms, the above point clouds are statistically analyzed, as shown in Table 3; the detection rate (Dr) is the percentage of bottom points with an error less than sqrt(0.3^2+^(0.015 × *d*)^2^) m (where *d* is the water depth); RMSE is the root mean square error of the bottom points; and max(*d*) denotes the detected maximum depth. Since there is an offset in dddNCFWF, its translation (dddNCFWF_T) that best matches the reference data is also considered in the following analysis.

The detection rates of Max and RLD_M are low, while ASDF_M shows a higher detection rate. The detection rate is significantly improved in ASDF_A, RLD_A and dddNCFWF. The detection rate of dddNCFWF is almost equal to ASDF_A but lower than RLD_A, because the fake signals with relatively high amplitude can be detected in dddNCFWF sometimes. In contrast, RLD can effectively keep the signal which is similar to the emitted waveform and remove other noise even with relatively high amplitude. The detection rate of QUAD is lower than RLD_A, indicating that the detection rate is decreased after waveform decomposition with the quadrilateral model. EFSP can improve the detection results in RLD_A with the highest detection rate of 76.64%. RMSE reflects the accuracy of the detected points. ASDF_M has the minimum RMSE, but its detection rate is too low. dddNCFWF has the largest RMSE. Although the accuracy can be improved by translation, there is still a large RMSE in dddNCFWF_T. The distance of the offset may be related to the signal strength, so it cannot be completely eliminated by the translation. Errors are introduced by the quadrilateral model with the RMSE increased from 0.1347 m to 0.1504 m in QUAD. After waveform decomposition with the EFSP model, the RMSE is reduced from 0.1347 m to 0.1076 m, indicating that EFSP may correct the initial value even if there is some error in it. EFSP has a great max(*d*), exceeding the traditional *maximum* method by 10 m. In addition, dddNCFWF has the maximum max(*d*) among the tested methods, implying that it may have a better performance with an appropriate filter.

#### 3.3.3. Adaptability Analysis of the EFSP Model

Since water depth is a key factor affecting the strength of the bottom signal, four waveforms in different depths are selected to test the adaptability of the model in waveform decomposition. The amplitudes of the bottom returns in these waveforms are 21.4, 3.2, 2.5, and 2 bins, respectively. For the first three waveforms, the initial values provided by RLD_A are correct, while the position of the bottom signal in RLD_A is wrong for the last waveform.

**Figure 21:**
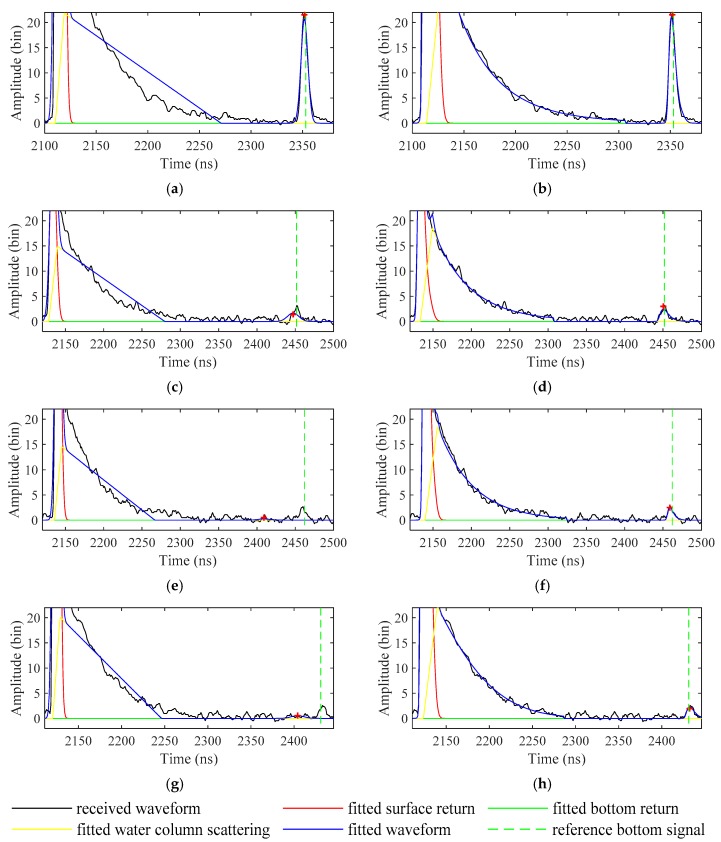
Waveforms fitted with (**a**,**c**,**e**,**g**) the quadrilateral model and (**b**,**d**,**f**,**h**) the EFSP model (zoom in the bottom). The peak of the fitted bottom return is marked by “+”.

The received waveforms and their fitting results are shown in Figure 21. It can be found that the quadrilateral model cannot fit water column scattering accurately compared with the EFSP model. When the bottom signal is fairly strong, the waveform decompositions with the two models can both converge to the exact position (see Figure 21a,b). With the decrease of bottom signal intensity, the inadaptability of the quadrilateral model becomes more and more obvious, even when the exact initial values are provided (see Figure 21c,e,g). In contrast, as long as the strengths of the bottom signals are sufficient, the locations of the bottom signals obtained with the EFSP model are precise regardless of the correctness of the initial values (see Figure 21d,f,h). 

### 3.4. Experiment Ⅳ: Accuracy Assessment for the Processing Algorithms

#### 3.4.1. Simulated Data

**Figure 22:**
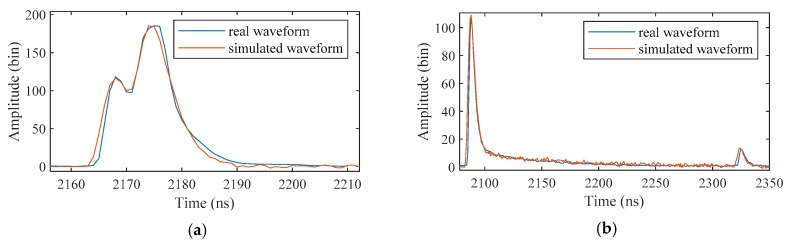
Comparison between the real waveforms and the simulated waveforms with depths of (**a**) 0.8 m and (**b**) 27 m.

To evaluate the accuracy of the surface and bottom signals, the water LiDAR waveform model (Wa-LiD) presented by Abdallah et al. [42] was applied in this experiment. Wa-LiD is a successful simulator for simulating green channel waveforms received from water, which has been widely used in ALB research [15,18,19]. It can perfectly reproduce the received waveforms by adjusting some realistic water parameters [42]. In this experiment, the Gaussian function in Wa-LiD was replaced by the calibration waveform to better fit the real received waveforms. As shown in Figure 22, the real waveforms can be well fitted by Wa-LiD with proper environmental parameters. However, there are some differences between the leading and falling edges of the signal, which may be induced by the slope of water surface and bottom.

To assess the accuracy of the bathymetric signals both in shallow and deep water, we generated 10,000 simulated waveforms in which the depth varied from 0 to 2 m with an interval of 0.0002 m, and 10,000 waveforms in which the depth varied from 40 m to 50 m with an interval of 0.001 m. The environmental parameters of each simulated waveform were randomly selected within the normal range which was determined by the real waveforms.

#### 3.4.2. Signal Detection in Shallow Water

For the simulated waveforms of shallow water (0–2 m), we test the waveform processing algorithms in Section 3.2.2. Since the accurate surface and bottom signal positions are known, the detection rate of surface (Dr_S) and bottom signal (Dr_B), and their RMSE (denoted by RMSE_S and RMSE_S, respectively) are assessed in Table 4. The accuracy of the surface signal is consistent with the field experiment results. EW still has the highest detection rate and accuracy. The Dr_S in simulated data is generally higher than that in field data, because the effect of waves on the water surface is not considered in the simulated waveforms. It is worth noting that the Dr_S of GD is higher than that of RLD_A, but the Dr_B of GD is significantly lower than that of RLD_A indicating that GD is not adaptive to the waveforms. Due to the changes of the environmental parameters, the Dr_S of RLD_A is lower than that of RLD_M. However, EW is still able to achieve the correct decomposition with inaccurate initial values, which depends on the appropriate model (EW) and a good optimization algorithm (TR).

#### 3.4.3. Signal Detection in Deep Water

The waveform processing methods in Section 3.3.2 are evaluated with the simulated waveforms of deep water (40–50 m), and the statistical results are shown in Table 5. By comparing Table 3 and Table 5, it can be found that the translation of dddNCFWF (dddNCFWF_T) performs better than EFSP here. Because the simulated waveform does not take into account the signal stretching caused by the slope of the water surface, the offset in dddNCFWF becomes a fixed value which can be eliminated by translation. It also proves that the error of dddNCFWF is mostly caused by the mismatch between the symmetric filter and the asymmetric emitted waveform. Apart from dddNCFWF_T, EFSP still has the best performance. Consistent with the results of the field experiment, the adaptive threshold significantly increases the Dr_B, and the Dr_B of RLD_A is still greater than that of ASDF_A or dddNCFWF, showing that RLD_A are the best initial values. Although EFSP does not increase the Dr_B of RLD_A, the RMSE_B of EFSP has been significantly improved. Even for the weak bottom signals in deep water, constructing a suitable model is also useful for improving the accuracy.

## 4. Conclusions

In this paper, a depth-adaptive waveform decomposition method for ALB was developed by classifying and fitting the waveforms in the green channel based on the similarities between them and the water column scattering. The application of the proposed method in shallow water datasets (where the depth is less than 2 m) and deep water datasets (with depths ranging from 20 m to 60 m) showed that this method can cope with most of the waveforms and significantly improves the accuracy of the detected signals. The main conclusions are as follows:

1. Water column scattering can be used as a sign to distinguish the received waveforms in terms of depth. The defined parameter *S* can be used to measure the similarity between the received waveforms and the water column scattering. Since water column scattering is covered in the shallow water waveform, the *S* of the shallow water waveform is obviously greater than that of the deep water waveform. Thus, waveforms can be classified precisely according to *S*.
2. For the waveform preprocessing, improving the signal resolution is more efficient than denoising. With an appropriate signal detection threshold, RLD always performs better than ASDF with a higher signal detection rate. Although filtering algorithms can remove the noise in signals and improve the accuracy of signal detection, the weak bottom signal may be filtered out as noise in the meantime.
3. The adaptive threshold can improve the reliability of the signal detection. The intensity of the bottom signal varies greatly with water depth, while the noise in water column scattering may be stronger than the bottom signal, leading to the detection of fake signals. Furthermore, although RLD is a deconvolution algorithm with good noise resistance, noise is inevitably introduced in the process. The adaptive threshold can better cope with the fake signals because it takes into account the effects of the water column.
4. With an appropriate model and reliable initial values, waveform decomposition can significantly improve the signal detection rate and accuracy. The proposed models, EW and EFSP, can fit the waveforms well in most cases. Compared with the Gaussian function, the transformation of the calibration waveform can better fit the water surface and bottom signals. The exponential function with a second-order polynomial is consistent with the shape of water column scattering in the waveform. The TR algorithm can solve the model parameters in a reasonable region and provide an accurate solution. The results of waveform decomposition are based on the whole waveform and are accurate to the sub-sampling interval. Even when the initial values are wrong, the detection results can be corrected by waveform decomposition in some cases. In addition, the processing time of waveform decomposition is long, meaning that whether the wave decomposition step should be added depends on the accuracy requirements in practical applications. The waveform decomposition model proposed in this paper is for the Mapper5000 system and may need relevant adjustments when applied to waveforms acquired by other ALB systems.

The applicability of the model is very important in waveform decomposition. In shallow water, due to the random fitting of water column scattering, a bias between the surface return fitted by EW and the surface signal detected from the NIR channel can be found. For future research, it would be useful to study the fitting of water column scattering in extremely shallow water. Using physical models rather than empirical models to fit water column scattering may be a breakthrough in this field.

## Figures and Tables

**Table 1:** Field data acquisition parameters.

Pulse Repetition Frequency	Sampling Speed	Beam Divergence	Altitude	Speed	Swath Width
5 kHz	1 GHz	NIR: 2.5 mrad green: 1 mrad	300 m	190 km/h	160 m

**Table 2:** Performance assessments for surface detection results with field data.

Algorithm	Dr (%)	RMSE (m)	min(*d*) (m)	Std.
Max	95.39	0.1645	0.4414	0.2276
RLD_M	97.53	0.1218	0.3292	0.1309
RLD_A	97.19	0.1085	0.3292	0.1107
GD	97.29	0.1174	0.2208	0.1456
EW	99.11	0.0901	0.2208	0.1159
Reference	58.54	—	—	0.0839

**Table 3:** Performance assessments for bottom detection results with field data.

Algorithm	Dr (%)	RMSE (m)	max(*d*) (m)
Max	31.22	0.1694	29.57
ASDF_M	53.80	0.0944	35.25
RLD_M	33.13	0.1498	30.36
ASDF_A	71.16	0.1124	37.50
RLD_A	73.88	0.1347	38.60
dddNCFWF	71.01	0.3798	41.14
dddNCFWF_T	71.98	0.2247	40.95
QUAD	66.39	0.1504	37.36
EFSP	74.64	0.1076	40.49

**Table 4:** Performance assessments for detection results in shallow water with simulated data.

Algorithm	Dr_S (%)	Dr_B (%)	RMSE_S (m)	RMSE_B (m)	min(*d*) (m)
Max	69.88	66.96	0.1433	0.6365	0.5920
RLD_M	76.81	74.95	0.1437	0.3686	0.4178
RLD_A	71.77	74.87	0.1311	0.1266	0.4180
GD	78.70	59.07	0.1697	0.8832	0.1540
EW	94.75	97.92	0.1059	0.0845	0.0558

**Table 5:** Performance assessments for detection results in deep water with simulated data.

Algorithm	Dr_S (%)	Dr_B (%)	RMSE_S (m)	RMSE_B (m)	max(*d*) (m)
Max	100	0.00	0.0469	—	—
ASDF_M	100	0.22	0.0508	0.0835	43.36
RLD_M	100	20.35	0.0857	0.1896	49.92
ASDF_A	100	42.29	0.0508	0.1012	49.70
RLD_A	100	56.34	0.0857	0.1702	49.92
dddNCFWF	100	52.29	0.3994	0.4217	49.92
dddNCFWF_T	100	59.61	0.0434	0.0850	49.92
QUAD	100	46.97	0.1416	0.2573	49.92
EFSP	100	56.69	0.0616	0.0681	49.92

**Figure A1:**
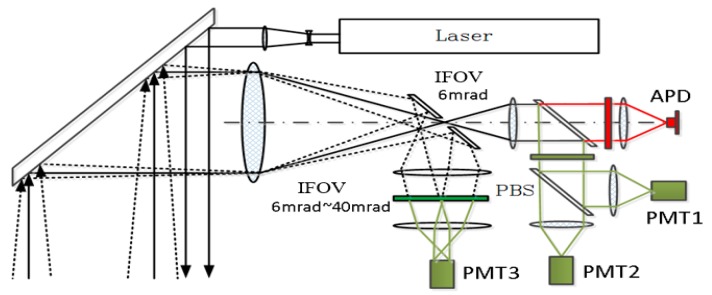
Schematic diagram of optical system in the Mapper5000 system [44]. IFOV: instantaneous field of view; APD: avalanche photodiode; PMT: photomultiplier tube; PBS: polarization beam splitter. The “Laser” contains a green laser and an NIR laser. The IFOV of the receiver is separated into two parts: a small center IFOV (6 mrad) and a large edge IFOV (6–40 mrad). The green light obtained from the small IFOV is split by a PBS and is received by PMT1 and PMT2.

**Figure A2:**
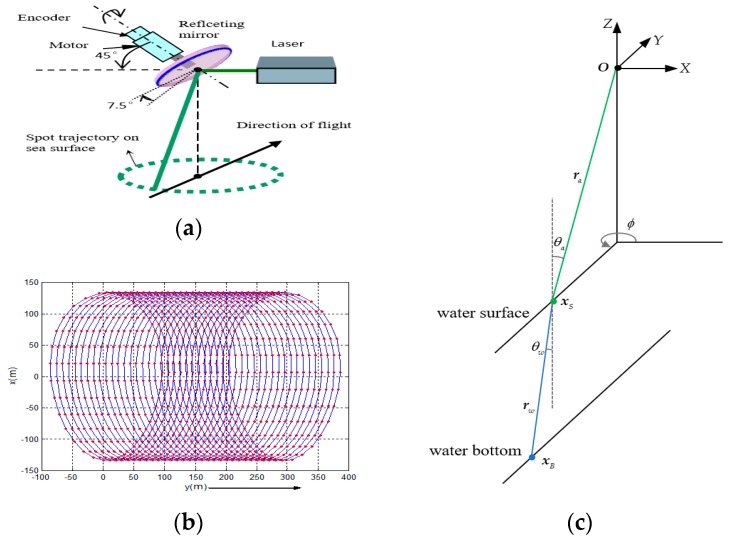
(**a**) The rotating scanner of Mapper5000 and (**b**) the spot trajectory on sea surface. The angle between the normal of the reflecting mirror and the axis of rotation is 7.5°, resulting in an elliptical spot trajectory on the sea surface [44]. (**c**) The mechanism of the refraction correction for Mapper5000. The ray path of the green laser in air and water are denoted by green and blue line, respectively.

## Appendix A

As shown in Figure A1, the NIR laser and the green laser share a scanner which produces an elliptical scan pattern, and the footprint has an almost elliptical shape at the sea surface [43,44]. With an altitude of 300 m, the footprint of the NIR laser is 0.8 m along-track and 0.78 m cross-track, and the footprint of the green laser is 0.32 m along-track and 0.31 m cross-track. The average point density on the sea bottom amounts to 0.8 points/m^2^. The IFOV of the receiver is divided into two parts: a small center IFOV (6 mrad) and a large edge IFOV (6–40 mrad). The APD, PMT1 and PMT2 channels use the small IFOV while the PMT3 channel uses the large IFOV. The sensor simultaneously collects full waveforms from four channels (NIR, PMT1–PMT3) with a sampling interval of 1 ns, corresponding to a distance of 0.15 m on the slant path in the air.

## Appendix B

As shown in Figure A2a, the scanner in the Mapper5000 system contains a rotating mirror which is controlled by a motor. The angle between the laser emission direction and the axis of rotation is 45°, and the angle between the normal of the reflecting mirror and the axis of rotation is 7.5°. As the mirror rotates, the laser will create an elliptical trajectory on the sea surface (see Figure A2b).

In this paper, the point clouds are generated by a data processing software which is designed for Mapper5000. After extracting the bathymetric signals from the waveform, a data file is generated containing the surface signal position *t_S_*, bottom signal position *t_B_*, the emitted signal position *t*_0_, the encoder data of the scanner and the GPS time of each waveform. The data file and the POS data are input into this software, and the point cloud data can be generated. The refraction correction is also completed in this software, the principle is as follows.

As shown in Figure A2c, a right-handed coordinate system is established with the laser as the origin, the flight direction as the positive direction of the *Y*-axis, and vertical upward direction as the positive direction of the *Z*-axis. According to the encoder data, the emitted direction of the green laser can be determined, including the angle *θ_a_* between the laser beam and the *Z*-axis, and the angle *φ* between the projection of the laser beam on the XY plane and the positive direction of the *X*-axis. Then the coordinates of the water surface point can be expressed as:

(21) $$\begin{array}{l} x_{S}=O+r_{a}\\ r_{a}=L_{a}\gamma_{a}=\frac{c_{a}\Delta t_{a}}{2}\gamma_{a}=\frac{c_{a}\left(t_{S}-t_{0}\right)}{2}\gamma_{a}\\ \gamma_{a}={\left(\sin\theta_{a}\cos\phi ,\sin\theta_{a}\sin\phi ,\cos\theta_{a}\right)}^{T}, \end{array}$$

where ***r****_a_* is the ray of the laser in the air equal to the product of its unit vector ***γ****_a_* and the transmission distance *L_a_*, *c**_a_* is the velocity of light in air, and Δ*t_a_* is the two-way transmission time in the air. According to the Snell’s law, when the laser pulse incident on the air-water interface the incidence angle will change from *θ_a_* to *θ_w_*, and the velocity of light will decrease from *c**_a_* to *c_w_*.

(22) $$\frac{\sin\theta_{a}}{\sin\theta_{w}}=\frac{n_{a}}{n_{w}}=\frac{c_{w}}{c_{a}},$$

where *n_a_* and *n_w_* are the refractive indexes of air and water equal to 1 and 1.33, respectively. The coordinates of the water bottom point are given by:

(23) $$\begin{array}{l} x_{B}=O+r_{a}+r_{w}\\ r_{w}=L_{w}\gamma_{w}=\frac{c_{w}\Delta t_{w}}{2}\gamma_{w}=\frac{c_{w}\left(t_{B}-t_{S}\right)}{2}\gamma_{w}\\ \gamma_{w}={\left(\sin\theta_{w}\cos\phi ,\sin\theta_{w}\sin\phi ,\cos\theta_{w}\right)}^{T}, \end{array}$$

where ***r****_w_* is the ray of the laser in the water equal to the product of its unit vector ***γ****_w_* and the transmission distance *L_w_*, and Δ*t_w_* is the two-way transmission time in the water. Since a POS system is mounted on the same platform as the laser, the instantaneous position and attitude of the platform in WGS84 can be known from the GPS time and POS data, and the coordinates of the water surface and bottom points can be converted to WGS84.
